# Abnormal Liver Function Induced by Space-Occupying Lesions Is Associated with Unfavorable Oncologic Outcome in Patients with Colorectal Cancer Liver Metastases

**DOI:** 10.1155/2018/9321270

**Published:** 2018-05-15

**Authors:** Zheng Jiang, Chunxiang Li, Zhixun Zhao, Zheng Liu, Xu Guan, Ming Yang, Xiaofu Li, Dawei Yuan, Songbo Qiu, Xishan Wang

**Affiliations:** ^1^Department of Colorectal Surgery, Cancer Institute & Hospital, Chinese Academy of Medical Sciences, Peking Union Medical College, Beijing 100021, China; ^2^Department of Thoracic Surgery, Cancer Institute & Hospital, Chinese Academy of Medical Sciences, Peking Union Medical College, Beijing 100021, China; ^3^Department of Colorectal Cancer Surgery, The 2nd Affiliated Hospital, Harbin Medical University, Harbin, Heilongjiang 150001, China; ^4^Department of Magnetic Resonance Imaging, The 2nd Affiliated Hospital, Harbin Medical University, Harbin, Heilongjiang 150001, China; ^5^Genesis (Beijing) Co., Ltd., Beijing 100102, China; ^6^Department of Experimental Therapeutics, The University of Texas MD Anderson Cancer Center, Houston, TX 77054, USA

## Abstract

An early prediction of prognosis for patients with colorectal liver metastasis (CRLM) may help us determine treatment strategies. Liver function reflects the effect of the overall metastatic burden. We investigated the prognostic value of liver function in CRLM patients. In our study, patients with abnormal LFTs (liver function tests) had a poorer prognosis than did those with normal LFTs (*P* < 0.05). A multivariate analysis revealed that LFTs was an independent prognostic factor for CRLM. For those patients with abnormal LFTs, novel prognostic contour maps were generated using LFTs, and no positive correlation exists between the values of survival duration and abnormal LFTs. Additionally, the MTVR (metastatic tumor volume ratio) was measured directly by magnetic resonance imaging and was shown to be highly correlated to LFTs by a Pearson correlation analysis. A multivariate logistic regression analysis also demonstrated that the MTVR and hepatectomy were independently predictive of abnormal LFTs. The space-occupying effect of metastatic lesions can cause abnormal LFTs, resulting in a poor prognosis. Biochemical analyses of LFTs at the initial diagnosis of CRLM enable the stratification of patients into low- and high-risk groups; it may help clinicians determine promising treatment strategies.

## 1. Introduction

More than 140,000 patients are diagnosed with colorectal cancer (CRC) each year in the United States [1]. Approximately 60% will develop liver metastases [2]. The prognosis for metastatic CRC has significantly improved in the past 10–15 years, with more effective surgical approaches and efficacious chemotherapy regimens making it possible for patients to undergo surgical resection [3]. Even though the vast majority of metastatic CRC patients (80%–90%) present with unresectable disease, modern combination chemotherapy results in a median survival duration of roughly 20 months [4–6]. However, evaluating the prognosis of patients with CRC liver metastasis (CRLM) is still challenging, and the results will influence treatment strategies.

The TNM Classification of Malignant Tumors is the main prognostic tool used in clinical practice. However, it is not sufficient to differentiate the likelihood of survival in stage IV cases. Therefore, new methods of predicting and improving outcome are being explored [7–10]. Numerous oncologists and clinical researchers have assessed the relevance of liver function tests (LFTs) for early detection of liver metastasis in patients with different types of cancer [11, 12]. However, the conclusions remain inconsistent [13, 14].

Although the role of LFTs in identifying metastases to the liver remains unclear, on the basis of our clinical experience, we speculated that abnormal LFTs might be useful for predicting prognosis in patients with CRLM, which is induced by space-occupying lesions. Therefore, in this retrospective study, we determined the prognostic value of LFTs in patients with a definitive diagnosis of CRLM.

## 2. Materials and Methods

### 2.1. Patients

After receiving approval from the institutional review board, we searched the patient data bank at our institution to identify all consecutive patients who underwent operative and conservative treatment for CRLM and were first seen between December 1987 and June 2010. For compatibility, only patients who met the following criteria were considered for further analysis: (1) age ≥ 18 years and ≤75 years; (2) none of the patients in either group had other known liver disease at entry into the study; (3) previous normal LFTs; (4) no evidence of extrahepatic metastases; (5) no history of cancer; (6) complete follow-up data; and (7) LFTs available for the date of diagnosis of CRLM. Most patients with resectable liver metastases from colorectal cancer have received operation within 1 week after initial diagnosis.

The diagnosis of liver metastasis was confirmed by fine needle aspiration biopsy or typical clinical and imaging findings, disease progression, and the absence of any additional cancer. Tumors were staged in accordance with the American Joint Committee on Cancer Staging System. For the survival analysis, progression-free survival was measured from the time of diagnosis to the time of tumor progression. Overall survival (OS) was defined as the time from the date of diagnosis of liver metastases to the date of patient death or last follow-up.

### 2.2. Biochemical Measurements

LFTs were measured in a core laboratory and were considered abnormal when levels exceeded 40 U/l for alanine transaminase (ALT), 40 U/l for aspartate transaminase (AST), 60 U/l for gamma glutamyltransferase (*γ*GT), 240 U/l for lactate dehydrogenase (LDH), and 150 U/l for phosphatase alkaline (AP). The blood was taken at inpatient for most patents with simultaneous hepatic metastases. 125 patients who developed liver metastases while undergoing regular (every 3 months) follow-up with LFTs and liver imaging were included in this study, and the blood was taken at outpatient clinic. The blood was taken at the initial diagnosis for CRLM, and the value was analyzed in this study.

The LFTs were classified as normal or abnormally elevated, according to the laboratory ranges. They were analyzed in isolation or were combined. Combined tests were analyzed using the following variables: 1 abnormal test result, 2 abnormal test results, 3 or 4 abnormal test results, or 5 abnormal test results.

### 2.3. Magnetic Resonance Imaging (MRI) Measurements

One hundred thirteen patients underwent multiphase liver MRI at the initial diagnosis of CRLM. MRI examinations were conducted using a 1.5 T system (Magnetom Symphony, Siemens, Erlangen, Germany). Volumetry on MRI was performed by one investigator (XFL) with 12 years' experience in abdominal MRI, supervised by an experienced hepatobiliary surgeon. After the imaging data had been transferred, the volume of the liver and lesions were measured using ImageJ, a software package for image analysis developed by the National Institutes of Health that can be freely downloaded from their website (https://rsb.info.nih.gov/ij/download.html).

All calculations using total liver volumes and metastatic tumor volumes (MTVs) were performed without liver remnant volumes. The MTV ratio (MTVR) was calculated as MTV/total liver volume.

### 2.4. Statistical Analyses

Patients' clinical characteristics are presented as means or medians for continuous variables and as percentages for categorical variables. Comparisons between normal and abnormal LFT groups for categorical variables were performed using Fisher's exact test or *χ*^2^ tests. Survival curves were constructed using the Kaplan-Meier method, and the univariate survival difference was determined using the log-rank test. Time-point survival was estimated using the life-table method. Adjusted hazard ratios (HRs) with 95% confidence intervals (CIs) were calculated using Cox proportional hazards models.

The correlation between MTVR and LFTs was measured using the Pearson's *R* correlation test. A multivariate logistic regression analysis was performed to determine the variables associated with abnormal LFTs. Statistical analyses were performed using Stata statistical software, version 10.0 (StataCorp). Two-sided *P* < 0.05 was considered statistically significant.

## 3. Results

### 3.1. Characteristics of the Study Population

Of 1337 patients with CRLM, 552 (349 men and 203 women) met the inclusion criteria and were considered for further analysis; their median age was 58 years (range: 22–75). Their demographic and primary tumor characteristics are summarized in Table 1. The primary tumor was located in the colon in 271 patients (49.1%) and the rectum in 281 patients (50.9%). Primary cancer resection was performed in 450 patients (81.5%). Two hundred eighty-six patients (51.8%) had positive lymph nodes, as determined by a pathologic analysis of the colorectal specimen. Hepatic metastases were diagnosed simultaneously in 427 patients and metachronously in 125 patients and developed a mean of 20.9 months after CRC resection. Among the 552 patients with hepatic metastases from CRC, 22 (4.0%) underwent metastasectomy and 79 (14.3%) underwent ablation. Chemotherapy was administered in 193 patients (35.0%), with 9 patients treated by hepatic artery infusion. Ten patients (1.8%) underwent radiotherapy, and 384 received best supportive care (69.6%). The minimum follow-up duration was 0 months, and the maximum was 213 months (mean ± standard deviation, 13.5 ± 15.6 months).

### 3.2. Survival Differences by LFTs

**Figure 1:**
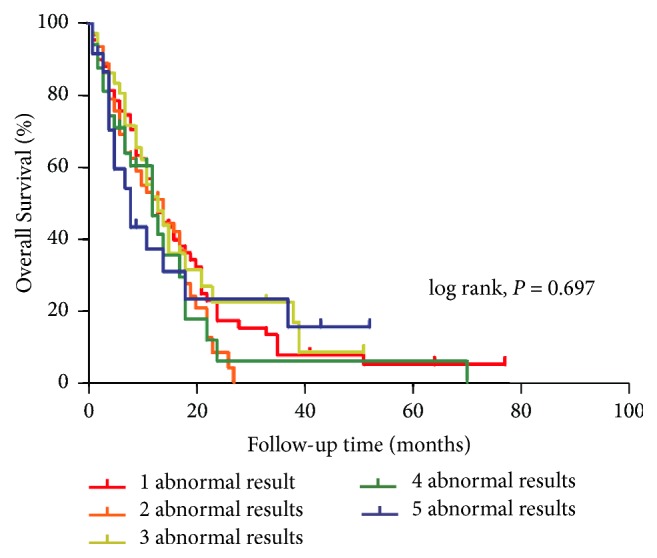
Kaplan-Meier curves showed no survival difference between the subgroups with isolated and combined variables.

**Figure 2:**
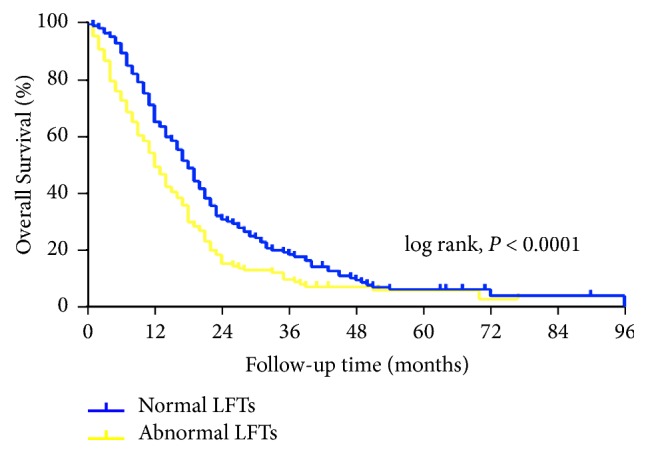
Kaplan-Meier curves showed significant survival difference between two subgroups in accordance with LFTs values.

**Figure 3:**
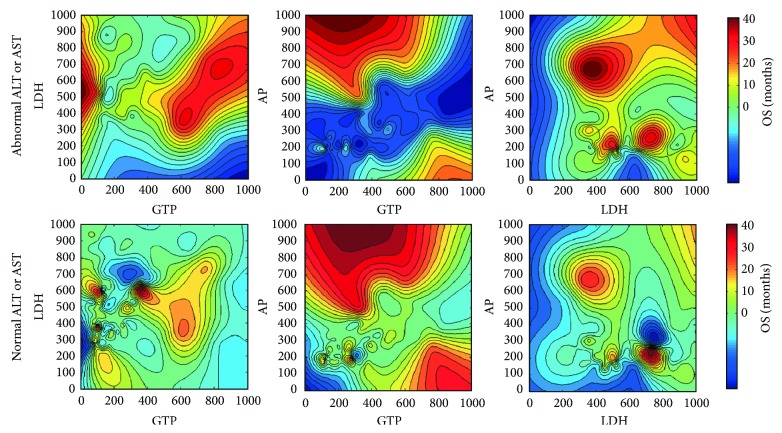
Contour maps for investigating the association between the values of survival duration and abnormal LFTs. Red areas depict favorable prognosis and blue areas unfavorable prognosis.

Table 2 displays the detailed survival characteristics according to the LFTs. Patients with abnormal LFTs had poorer OS and progression-free survival durations than did those with normal LFTs. Among patients with abnormal values, those with elevated LDH levels had the poorest prognosis, with a median survival duration of only 9 months. The 2-year survival rates of the five subgroups (normal, AP, *γ*GT, LDH, and ALT and/or AST) were 31.0%, 20.0%, 17.8%, 8.0%, and 16.8%, respectively (*P* < 0.05). When the combined LFTs were analyzed, however, there was no significant difference in prognosis between the patients with isolated and combined variables (Figure 1). Therefore, we divided patients into two subgroups—abnormal and normal LFTs—and performed a survival analysis. As shown in Figure 2, there were significant differences in OS between the two subgroups (*P* < 0.05). The median survival durations of the abnormal and normal LFT subgroups were 12 and 18 months, respectively. On multivariate analysis using the Cox proportional hazard model, LFTs were also an independent prognostic factor for CRLM (*P* = 0.0001; HR with 95% CI: 1.52 [1.22–1.88]). Subsequently, for revealing the relationships between the values of LFTs and survival time, the prognostic contour maps were produced using abnormal LFTs values, which indicated the probability of outcome (Figure 3). We found that no positive correlation exists between the values of survival duration and abnormal LFTs.

### 3.3. A High MTVR Was Associated with Abnormal LFTs

Table 2 shows that LFTs were associated with marked clinical characteristics. Tumor size, number of liver metastases, progression-free survival, OS, hepatectomy or ablation history, and chemotherapy history were significantly associated with abnormal LFTs (*P* < 0.05). Larger number or size of liver metastatic tumors was the indicator of abnormal LFTs. We speculated that LFTs reflect the combined effect of diminished liver function and the overall metastatic burden.

**Figure 4:**
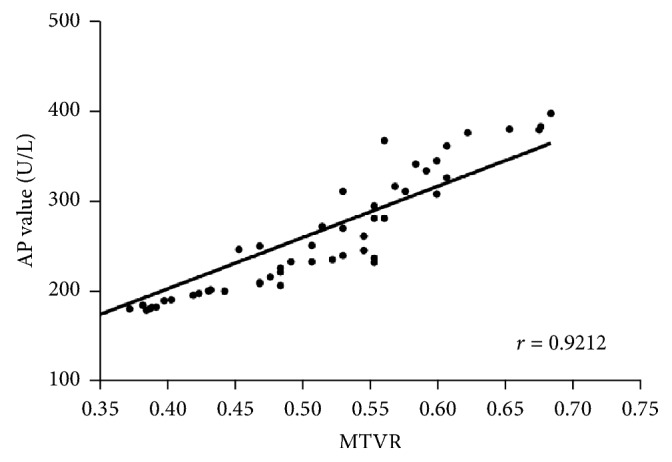
Correlation between MTVR and AP value.

The MTVR, which reflects the tumor burden, is initially expressed as a ratio of the metastatic tumor volume to the total liver volume, as measured directly by MRI. MTVR values were measured by retrospectively analyzing diagnostic MRI scans in the 113 CRLM patients for whom MRI data were available. A Pearson correlation analysis revealed that the MTVR was highly correlated with LFTs, such as phosphatase alkaline (Pearson correlation coefficient, 0.92) (Figure 4). We used a multivariate logistic regression analysis to estimate the parameters of a qualitative response model. Only the MTVR (*P* < 0.001, HR with 95% CI: 2.532 [1.410–4.545]) and hepatectomy (*P* = 0.009, HR with 95% CI: 3.448 [1.209–9.901]) had independent predictive value.

## 4. Discussion

Worldwide, CRC is the second most commonly diagnosed cancer in women and the third in men, with over 1.2 million new cases and 608,700 deaths yearly [15, 16]. The liver is a common site of tumor spread, and in approximately 30% of cases, synchronous liver disease is present at the time of diagnosis. A further 50% of patients develop CRLM during the course of their illness [17]. Aggressive metastasectomy, combined with advancements in systemic chemotherapy, has led to significantly improved outcomes in patients with CRLM [18–20]. The overall 5-year survival rate for patients with resectable CRLM isolated to the liver is between 35% and 60% [21, 22] and the median survival duration is up to 22 months with systemic chemotherapy alone [23–25]. Unfortunately, the vast majority (75%–80%) of patients with CRLM are deemed unsuitable for surgical resection at initial diagnosis [26, 27]; thus, there remains a high demand for effective CRLM treatments and improved palliative results. For longer life expectancy and better quality of life, evaluating the prognosis of CRLM patients is extremely important in clinical routine, and it may help us determine promising treatment strategies.

LFTs likely reflect the combined effect of diminished liver function and the overall metastatic burden. Certain types of agents used for chemotherapy, such as irinotecan, oxaliplatin, and 5-fluorouracil, can cause hepatic damage [28–30]. Most CRC patients in our study underwent adjuvant chemotherapy to decrease the risk of recurrence. To eliminate the effect of chemotherapy or other factors, only the patients with previously normal LFTs were included. We found that patients with abnormal LFTs had poorer outcomes than did those with normal LFTs on both univariate and multivariate survival analysis. We hypothesized that abnormal LFTs, which are caused by “space-occupying effect” of metastatic liver lesions, are associated with poor prognosis. For major hepatectomy, 30% of the total liver volume is considered to be sufficient to maintain adequate liver function [31]. In other words, up to 70% of the liver can be resected, including metastatic cancer and paracancerous and noncancerous tissue. Moreover, preexisting liver lesions may be underestimated on the basis of MRI volumetric studies. All of the above reasons may explain why some abnormal LFT values were associated with low MTVRs in this study (Figure 4). Although we found that the patients with abnormal LFTs had a poorer prognosis based on current data, no positive correlation between the values of survival duration and abnormal LFTs was observed in further analysis (Figure 3), which suggested caution in the exploration of treatment strategies for these patients.

MTVR serves as an alternative to two factors, tumor size and number of liver metastases in easier assessment of “space-occupying effect.” Imaging plays an integral role in monitoring the status of CRLM. A variety of imaging techniques, including ultrasonography, computed tomography, and MRI, are used. MRI offers superior soft tissue resolution; therefore, it has several advantages over computed tomography in tissue characterization and the evaluation of background liver parenchyma. Small lesions can be detected and characterized more confidently with MRI [32, 33]. Therefore, in this study, we used MRI to measure MTVR. To verify the relevance of MTVR to abnormal LFTs, we used Pearson's *R* correlation test. We found that the value of LFTs increased with the MTVR value. Furthermore, on multivariate logistic regression analysis, MTVR and hepatectomy had independent predictive value.

Posthepatectomy liver failure remains an important cause of morbidity and mortality after major liver resection [34, 35]. Conventional biochemical LFTs, as evaluated by a routine blood test analysis, remain widely used and form an indispensable part of most definitions of liver failure [36, 37]. Undergoing liver resection results in postoperative changes in LFTs, which is consistent with our findings. Moreover, Grąt et al. recently found that LFTs on postoperative day 1 after major CLRM resection were substantially associated with outcome [38].

Our study has several limitations. First, most of the patients did not undergo hepatectomy for liver metastases. At the time many of these patients were diagnosed with CRLM, there were numerous barriers to obtaining surgical treatment, including a lack of experienced surgeons and factors such as a lack of health insurance, long travel distances, low health literacy, low education levels, and language barriers that affect patients' ability to navigate the medical system. Second, only some patients underwent MRI to determine the MTVR. MRI was used as a part of a routine diagnostic imaging procedure after 1998 at our hospital. In addition, imaging data of partial patients who underwent MRI were not available.

On the basis of our findings, we conclude that abnormal LFTs, which are induced not only by treatment factors but also by the space-occupying effects of metastatic lesions, will allow us to stratify CRLM patients into poor- and good-prognosis groups. It might help clinicians determine promising treatment strategies. However, a methodic and prospective study is needed to confirm these results, especially in high-risk patients selected by molecular analysis.

## Figures and Tables

**Table 1:** Demographic and primary tumor characteristics.

Clinicopathological features	Number (%)
Number of patients	552
Median age at diagnosis (range)	58 (22–87)
Gender	
Female	203 (36.8)
Male	349 (63.2)
Age	
≤60 years	313 (56.7)
>60 years	239 (43.3)
Location	
Rectum	281 (50.9)
Colon	271 (49.1)
Differentiation	
Well	13 (2.4)
Moderate	331 (60.0)
Poor	208 (37.7)
Mucinous histology	
Yes	36 (6.5)
No	516 (93.5)
T classification^&^	
T1/T2	24 (4.3)
T3/T4	368 (66.7)
Unknown	58 (12.9)
N classification^&^	
N0	160 (29.0)
N1	139 (25.2)
N2	147 (26.6)
Unknown	4 (0.7)
Perioperative chemotherapy	
Yes	397 (71.9)
No	155 (28.1)
Perioperative radiotherapy	
Yes	341 (61.8)
No	211 (38.2)
Resection margin^*∗*^	
R0	427 (77.4)
R1	23 (4.2)

^*∗*^Defined by findings on final pathological analysis (microscopic and major). ^&^American Joint Committee on Cancer Staging System.

**Table 2:** Colorectal liver metastases, treatment, and survival characteristics depending on normal/abnormal values of liver function tests.

Clinicopathological features	Normal	Abnormal AP	*P* value^*∗*^	Abnormal *γ*GT	*P* value^*∗*^	Abnormal LDH	*P* value^*∗*^	Abnormal ALT and/or AST	*P* value^*∗*^
No. patients	257	92		204		160		155	
Largest tumor size (cm)									
≤5	153	21		77		54		58	
>5	20	27	**<0.001**	53	**<0.001**	49	**<0.001**	40	**<0.001**
Number of liver metastases									
1	65	13		37		26		20	
>1	166	73	**0.017**	157	**0.029**	128	**0.011**	125	**0.001**
Hepatectomy									
Wedge resection	14	1		2		2		2	
Segmentectomy	2	0		1		0		1	
Hemihepatectomy	0	0		1		0		0	
No	241	91	**0.049**	200	**0.026**	158	**0.015**	152	**0.044**
Type of ablation									
RFA	26	11		34		16		26	
Cryotherapy	6	0		1		1		1	
No	225	81	0.901	169	0.155	143	0.573	88	**0.007**
Chemotherapy									
Yes	81	34		71		57		64	
No	176	58	0.341	133	0.456	103	0.386	91	**0.044**
MoAbs									
Yes	2	1		2		0		0	
No	255	91	0.863	202	0.816	160	0.263	155	0.271
HAIP placement									
Yes	4	2		4		4		3	
No	253	90	0.696	200	0.741	156	0.495	152	0.773
Radiotherapy									
Yes	7	0		2		1		1	
No	250	92	0.110	202	0.179	159	0.129	154	0.139
Progression-free survival (months)									
≤12	225	72		165		129		118	
>12	27	17	**0.042**	33	0.065	27	0.056	34	**0.002**
Overall survival									
median (months)	18	10		14		9		12	
Hazard ratio (95% CI)	-	0.57 (0.33–0.70)		0.68 (0.51–0.83)		0.50 (0.32–0.56)		0.62 (0.40–0.76)	
*P* value (log-rank)	-	**<0.001**		**<0.001**		**<0.001**		**<0.001**	
Survival rate (%) (95% CI)									
3 months	96.6 (93.3–98.3)	83.5 (72.8–90.3)		85.7 (79.5–90.0)		84.8 (77.6–89.8)		88.7 (81.9–93.0)	
6 months	89.6 (84.9–92.9)	64.2 (51.6–74.2)		71.7 (64.3–77.9)		65.8 (57.1–73.2)		78.3 (70.2–84.5)	
12 months	65.4 (58.5–71.5)	42.5 (30.1–54.3)		51.5 (43.3–59.0)		36.7 (28.1–45.2)		55.7 (46.2–64.3)	
24 months	31.0 (24.0–38.2)	20.0 (9.8–32.8)		17.8 (11.2–25.8)		8.0 (3.6–14.6)		16.8 (9.1–26.5)	

^*∗*^Compare with normal group. RFA, radiofrequency ablation; HAIP, hepatic artery infusion pump; MoAbs, monoclonal antibodies; CI, confidence intervals.
